# Unmasking and tackling the underestimation of the cholera burden in Africa: A viewpoint

**DOI:** 10.1371/journal.pntd.0013128

**Published:** 2025-06-05

**Authors:** Olushayo Oluseun Olu, Sylvester Maleghemi, Adebola Olayinka, Argata Guracha Guyo, Solomon Fisseha Woldetsadik, Ida-Marie Ameda, Chinwe Iwu-Jaja, Amos Petu, Abdulmumini Usman

**Affiliations:** 1 World Health Organization Regional Office for Africa, Brazzaville, Congo; 2 World Health Organization Headquarters, Geneva, Switzerland; 3 World Health Organization, Accra, Ghana; 4 World Health Organization Regional Emergency Hub, Nairobi, Kenya; 5 United Nations Children’s Fund Headquarters in Nairobi, Nairobi, Kenya; University of Oxford, UNITED KINGDOM OF GREAT BRITAIN AND NORTHERN IRELAND

## Abstract

Cholera remains a significant public health challenge in Africa, with the continent recording the highest Case Fatality Ratio of 1.9% among all regions from 2014 to 2023. Despite ongoing efforts, the true burden of cholera is substantially underestimated due to poor quality and incomplete data. This article aims to review the factors contributing to the underestimation of cholera in Africa and explore potential solutions to better characterize the disease epidemiology and burden on the continent. We drew on our field experiences and existing literature to identify the key factors responsible for cholera underestimation in Africa. We also propose strategies to improve cholera surveillance and reporting. We identified several factors contributing to cholera underestimation, including weaknesses in the Integrated Disease Surveillance and Response system, insecurity due to conflict situations, limited healthcare access, and the politicization of cholera outbreak data. We propose a comprehensive approach to address these challenges, including strengthening disease surveillance, adopting digital technologies to improve data collection and management, improving healthcare access, increasing public awareness and enhancing community engagement and participation in cholera reporting, and fostering political commitment to transparent data reporting. We urge African ministries of health and public health stakeholders to increase their commitment to and investment in strengthening cholera data management on the continent.

## Author summary

Cholera is a severe diarrheal disease caused by drinking or eating food contaminated with a bacteria called *Vibrio cholerae*. The disease is most common in Asia and Africa. The seventh cholera outbreak, which reached Africa in the 1970s, is still ongoing, with frequent and recurrent outbreaks in many countries. A major reason cholera keeps coming back is that the true number of cases is not accurately reported. This makes it difficult to plan and deliver effective responses, such as improving water and sanitation, sharing important health information, forecasting vaccine needs, and allocating resources. To solve this problem, we need better ways to collect and manage cholera data, use modern technology for tracking cases, improve access to healthcare, raise public awareness, and encourage governments to share cholera data openly. African health ministries and public health organizations should increase their efforts and investment in strengthening cholera data management across the continent.

## Introduction

**Fig 1:**
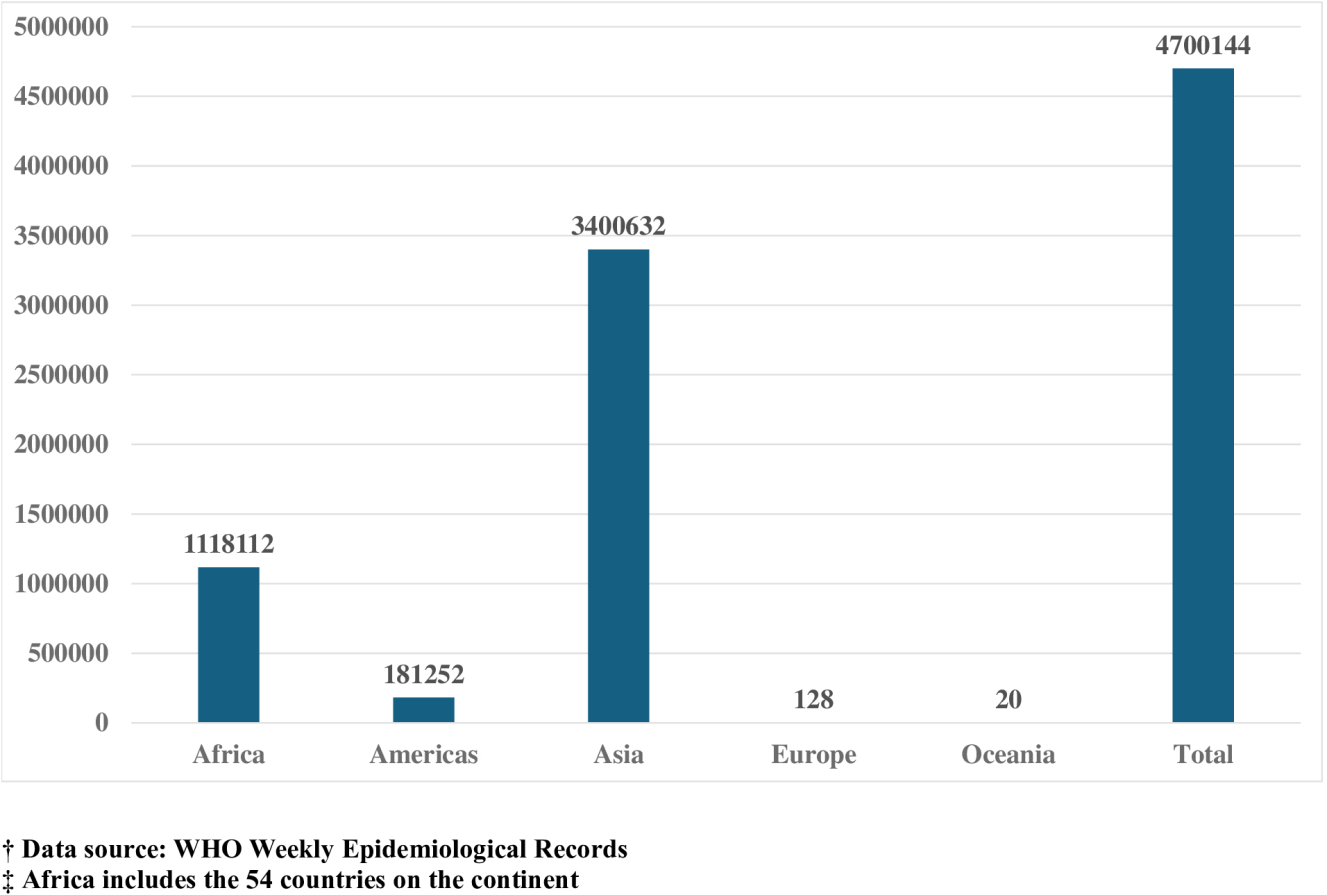
Global cumulative cholera cases from 2014-2023.

Despite ongoing efforts to combat the global cholera scourge [1,2], the disease remains a significant public health challenge, imposing a substantial social and economic burden on the world’s population [3]. Asia and Africa bear the greatest impact, accounting for 72% (3,400,632) and 24% (1,118,112), respectively, of the cumulative 4,700,144 cholera cases reported to the World Health Organization (WHO) from 2014 to 2023 (Fig 1). Notably, Africa recorded the highest Case Fatality Ratio of 1.9% among all the regions during this period. The seventh cholera pandemic, which reached Africa in the early seventies, continues to persist with recurrent epidemic waves affecting several countries [4]. Between January 2022 and July 2024, almost 400,000 cholera cases and over 7,000 deaths were reported from 18 African countries with the Democratic Republic of the Congo, Ethiopia, Mozambique, Malawi, Nigeria, and Zimbabwe accounting for more than 80% of the caseload and deaths [5].

The available evidence indicates that these reported cholera cases and deaths in Africa represent only a small portion of the true numbers, with estimates pointing to substantial underreporting [6–8]. For instance, Ali and colleagues estimated that the 331,337 cholera cases reported by WHO from 2008 to 2012 represented only 11.6% of the estimated number of cases, while the 6,335 reported deaths accounted for only 6.6% of the estimated fatalities [9]. Similarly, O-Tipo Shikanga and colleagues identified 46% more cholera cases and 200% more deaths than what were officially reported during a 2008 outbreak in western Kenya through active community case-finding [10]. Besides the data underestimation, poor quality, and incomplete data remain major hindrances to the detailed characterization of cholera epidemics on the continent [11]. The field experiences of the authors working in the prevention and control of several outbreaks on the continent also corroborate these findings. Comparable underestimations have also been documented in cholera-endemic regions outside of Africa, particularly in the aftermath of natural disasters and large-scale population displacement [7]. For example, following the 2004 flooding in Dhaka, Bangladesh, it is estimated that approximately 22% (3,740 cases) of the reported diarrhoeal disease cases were likely attributable to cholera, yet were not reported to WHO [7]. However, Sauvageot and colleagues suggested that cholera cases might also be overestimated in some African countries due to the limitations of syndromic surveillance and non-specific cholera case definitions [12]. This stands in contrast to the preceding discourse and further highlights the critical need for reliable estimates of cholera cases and deaths.

A precise understanding of the true burden of cholera including its morbidity, mortality, social, and economic impact is critical for effectively addressing the burgeoning disease trend [7]. Although several authors have reviewed the causes and effects of cholera underestimation globally, few have focused on Africa’s unique health system and social and economic context [7,13]. Likewise, very few have proposed context-specific solutions that are suited to the African setting. In this article, we review the factors contributing to the underestimation of cholera in Africa and explore potential solutions. This is with a view to contributing to better characterization of the disease epidemiology and management in Africa.

## Cholera underestimation as a driver of the chronic cholera situation in Africa

We believe that the inaccurate estimation of cholera cases in Africa plays a significant role in sustaining the ongoing transmission of the disease across the continent [14]. This occurs in several ways. First, incomplete and poor-quality data hinders a comprehensive epidemiological characterization of cholera outbreaks. This, in turn, hampers the effective targeting of response interventions such as water and sanitation improvements, risk communication, and vaccine forecasting, allocation, and distribution [13]. For instance, incomplete cholera line lists, a primary tool for outbreak data collection, often make in-depth descriptive and analytical descriptions of epidemics difficult [12]. This, in turn, poses challenges in delivering tailored interventions, especially for specific populations such as young and malnourished children, displaced and migrant populations, and pregnant women who are at high risk for severe cholera [15].

Second, inadequate characterization of the disease impairs strategic planning which results in poor allocation of resources for cholera risk reduction, prevention, and preparedness. As a result, outbreaks continue to recur, further exacerbating the public health burden [13]. Third, poor-quality epidemiological data can lead to inaccurate estimations of cholera’s economic burden, limiting effective advocacy, resource mobilization, and appropriate funding allocation for prevention and control. For example, Mogasale and colleagues highlighted significant disparities in the estimated versus actual economic burden of cholera in Africa, emphasizing the impact of underreporting on resource prioritization [3]. Last, underestimating the cholera burden may hinder the dissemination of accurate preventive information to affected populations and travelers, contributing to low-risk perception and reducing the effectiveness of public health advisories and interventions [7]. This not only increases the risk of transmission but also weakens community trust in health authorities and response efforts. The importance of addressing the cholera data gaps in Africa cannot therefore be overemphasized.

## Factors responsible for cholera underestimation in Africa

Traditionally, cholera surveillance in Africa is conducted within the framework of the Integrated Disease Surveillance and Response (IDSR) system, where the disease is designated as one of the epidemic-prone diseases that are immediately notifiable to WHO if detected within the routine or event-based surveillance systems [16]. Detection of a suspected case of the disease triggers immediate verification, case and field investigation to confirm the case and initiate the appropriate response. Confirmation of an outbreak activates the collection of additional data from the Cholera Treatment Centres (CTCs) and other health facilities using line lists.

However, a major limitation of this system lies in its inherent weaknesses, which present significant challenges in accurately estimating the cholera burden in Africa. While several studies have noted improvements in the IDSR’s ability to detect and respond to outbreaks over the past decade, numerous persistent challenges continue to affect its effectiveness [6,12,17–20]. These studies have highlighted key issues, including inadequate data completeness and timeliness, limited use of innovative data collection and analyses technologies, weak laboratory capacity, inadequate financing for IDSR activities, and insufficient staffing with inadequate skills and motivation. For example, as of 2017, only 23 (49%) out of the 47 countries in the WHO African Region had achieved the recommended threshold of over 80% for both timeliness and completeness of IDSR reporting [20]. Additional challenges include poor coordination and integration of data from various sources. For example, our field observations during several cholera outbreaks across the continent indicated that data from CTCs are often not fully integrated into routine IDSR data, leading to incomplete cholera records. Moreover, active community-based cholera surveillance and verbal autopsies, critical for identifying community cases and deaths, are infrequently implemented due to various constraints such as inadequate staffing, financial limitations, and logistical challenges in accessing remote areas.

Conflict situations, which are common across the continent, further hinder effective cholera surveillance due to the associated insecurity, poor infrastructure, and disrupted health systems, including attrition in the disease notification and monitoring systems and displacement of health workers including community health workers [21]. More than half (12) of the 22 countries classified by the World Bank as fragile and conflict-affected in 2025 are in Africa [22]. The majority of them (66%) continued to report significant numbers of cholera cases and deaths over the past decade. While an early warning system called the Early Warning Alert and Response Network (EWARN) has been introduced to address this challenge in most of these countries, they are often limited to secure and accessible areas [23]. Furthermore, access to healthcare services remains a major challenge in Africa, particularly in conflict-affected settings. According to WHO estimates, Africa’s healthcare index stands at just 0.32, indicating that the continent’s health systems can guarantee only 32% access to healthcare. In some countries, such as the Central African Republic, this figure drops as low as 12 [24]. With such limited healthcare access, many suspected cholera cases never reach health facilities and are therefore missed by formal surveillance systems. This challenge is further aggravated by a general lack of public awareness about cholera surveillance efforts [25], stigma [26], and mistrust of local health authorities and the services that they provide [27] leading to the concealment of cholera cases and deaths [28]. Given the inequitable access to formal health services across many parts of Africa [24], populations frequently seek care from informal providers, including private practitioners, traditional healers, and spiritual entities. These informal care providers usually operate independently of the formal health care system and typically lack established mechanisms for disease case reporting, thereby resulting in underreporting to the IDSR system.

The politicization of cholera outbreaks also contributes to the underreporting of cholera across Africa. National authorities often conceal outbreaks or underreport data when outbreaks are declared for a few reasons including concerns over potential travel and trade restrictions, which could have negative economic consequences [29]. Potential travel restrictions may also threaten the hosting of major regional political or sporting events with unpalatable political consequences. In addition, the stigma associated with being perceived as a country or community with poor access to water and sanitation further discourages transparent cholera reporting [13].

## Moving forward: Strategies to improve cholera surveillance and reporting in Africa

Building on the preceding discourse and drawing from the authors’ field experiences in cholera outbreak management across Africa, which was buttressed with existing scientific literature (S1 File), we propose a few recommendations for national health authorities and their partners to improve cholera data collection and integration and the estimation of the cholera burden on the continent.

First, cholera surveillance should be strengthened within the broader frameworks of the IDSR, EWARN, and the 2005 International Health Regulations (IHR 2005) in collaboration with regional public health organizations. This collaboration should focus on enhancing laboratory capacity for timely cholera diagnosis, particularly in peripheral health facilities and CTCs. Furthermore, refining cholera case definitions to improve their sensitivity and specificity, provision of standardized data collection tools and recruitment, continuous training, and supervision of data record officers and sustainable financing of IDSR activities at the health facility level are essential for improving case detection and surveillance accuracy. In addition, the implementation of mechanisms to ensure the completeness and timeliness of cholera line list reporting, as well as the integration of line list data with IDSR systems, is vital. The establishment of active community-based surveillance and case detection systems, coupled with the ongoing training and supervision of community health workers in active cholera surveillance, will further strengthen timely and comprehensive data collection during outbreaks.

Second, digital and innovative technologies, including artificial intelligence (AI) and digital health tools, should be systematically adopted and scaled up to improve all the phases of cholera surveillance from data collection, harmonization, analysis, through estimation of national and continental cholera trends. AI technologies could be used to improve both syndromic and genomic surveillance systems, enabling the rapid identification of novel *Vibrio cholerae* variants and supporting the timely development of diagnostic and therapeutic tools [30,31]. In addition, AI-driven tools such as predictive modeling and machine learning can combine diverse datasets like epidemiological, meteorological, and environmental information to more accurately forecast cholera outbreaks and transmission dynamics [30]. AI can also be applied to monitor and mitigate misinformation related to cholera, facilitating the delivery of timely, evidence-based public health communication to enhance risk communication and community engagement which could increase reporting [30]. In addition, AI technologies can contribute to improved data quality by increasing the accuracy, timeliness, and cost-efficiency of cholera data collection and reporting systems [30].

Third, the integration of social sciences and transdisciplinary operational research into more traditional cholera epidemiology will offer the opportunity to test multiple theories, perspectives, and factors influencing outbreak dynamics, and a way to optimally identify and address what can quickly become complex bottlenecks to response. Specifically, social science research should be employed to unravel the factors that shape societal beliefs, perceptions, and behaviors in response to cholera outbreaks, including the determinants of individuals’ and community’s willingness to report cases and deaths [32].

Fourth, diplomatically engaging national health authorities and political leaders proactively, under the leadership of African public health stakeholders, to encourage the effective collection and timely release of cholera and other epidemic data is imperative. This should be facilitated by ensuring data security and confidentiality, strengthening advocacy efforts, and providing incentives to encourage the sharing of transparent and accurate data [33]. Fifth, community engagement and awareness about cholera, emphasizing the importance of prevention, early treatment seeking, and reporting all cases and deaths through appropriate channels, including at the community level, should be scaled up. This should be guided by the findings of social science research and integrated into broader outbreak risk communication and community engagement strategies, including community monitoring and feedback mechanisms. Implementation of these strategies will not only enhance the utilization of cholera services which would by extension improve the capturing of cases and deaths but also strengthen active community-based surveillance and increase community trust in cholera services and reporting. Furthermore, to address the challenge of underreporting from the informal health sector, national and sub-national health authorities should establish structured mechanisms for engaging and incorporating informal healthcare providers into cholera surveillance systems. This can be achieved by developing simplified reporting tools tailored to their capacities, providing targeted training on cholera and other priority disease recognition and notification procedures, and linking them to the IDSR and community-based surveillance systems.

Sixth, in conflict-affected settings, the EWARN systems should be expanded using existing community-based health initiatives. A notable example is South Sudan’s Boma Health Initiative, which has a component of community-led disease surveillance [34]. Seventh, cholera stakeholders across Africa, including regional and non-governmental public health organizations, should continue to explore partnership opportunities to jointly support national and sub-national health authorities in strengthening cholera surveillance and data management capacities. Additionally, these organizations should collaborate to strengthen and harmonize the various cholera data collection systems, some of which are independently managed by different stakeholders, to ensure consistency and reliability.

Last, the National Action Plans for Health Security (NAPHS) or its equivalent, which have been developed by several African countries, provide a sustainable platform to accelerate the implementation of the foregoing recommendations. We recommend the costing, funding, and implementation of these plans [35]. Furthermore, future research to evaluate the impact of improved cholera reporting on key disease outcomes, such as time to outbreak containment, case fatality rates, and the effectiveness of outbreak response interventions would be essential. We call for careful documentation of such research and sharing of the lessons learned and successful strategies that can be replicated in different settings.

## Conclusion

The underestimation of the cholera burden in Africa is a complex challenge driven by weak surveillance systems, conflicts, economic and political barriers, stigma, and inadequate community awareness about the importance of reporting cholera cases and deaths. This, we believe, contributes to the sustained transmission of the disease on the continent. Addressing these challenges will require a comprehensive approach within the broader framework of IDSR, IHR 2005, EWARN, and NAPHS. This should include the implementation of interventions aimed at strengthening disease surveillance systems, increasing access to healthcare, improving community awareness, and fostering political commitment to transparently report cholera data. We believe that these would facilitate a better understanding of the true disease burden and ensure more effective disease prevention and control, ultimately saving lives and improving public health outcomes. We urge African ministries of health and public health stakeholders to increase their commitment to and investment in strengthening cholera data management on the continent.

## Supporting information

S1 File Literature search strategy. (DOCX)

## References

[1] Global Taskforce on Cholera Control. Ending cholera: a global roadmap to 2030. 2017. https://www.gtfcc.org/about-cholera/roadmap-2030/

[2] Kapaya F, Keita M, Sodjinou VD, Nanyunja M, Mpairwe A, Daniel EO, et al. An assessment of the progress made in the implementation of the regional framework for cholera prevention and control in the WHO African region. BMJ Glob Health. 2025;10(1):e016168. doi: 10.1136/bmjgh-2024-016168 39848635 PMC11759201

[3] Mogasale V, Ngogoyo SM, Mogasale VV. Model-based estimation of the economic burden of cholera in Africa. BMJ Open. 2021;11(3):e044615. doi: 10.1136/bmjopen-2020-044615 33757949 PMC7993295

[4] Mintz ED, Tauxe RV. Cholera in Africa: a closer look and a time for action. J Infect Dis. 2013;208 Suppl 1:S4-7. doi: 10.1093/infdis/jit205 24101644

[5] World Health Organization. Cholera in the WHO African Region. AFRO-Cholera Monthly Bulletin-July 2024.pdf

[6] Koua EL, Moussana FH, Sodjinou VD, Kambale F, Kimenyi JP, Diallo S, et al. Exploring the burden of cholera in the WHO African region: patterns and trends from 2000 to 2023 cholera outbreak data. BMJ Glob Health. 2025;10(1):e016491. doi: 10.1136/bmjgh-2024-016491 39848637 PMC11891530

[7] Zuckerman JN, Rombo L, Fisch A. The true burden and risk of cholera: implications for prevention and control. Lancet Infect Dis. 2007;7(8):521–30. doi: 10.1016/S1473-3099(07)70138-X 17584531

[8] Mengel MA, Delrieu I, Heyerdahl L, Gessner BD. Cholera outbreaks in Africa. Curr Top Microbiol Immunol. 2014;379:117–44. doi: 10.1007/82_2014_369 24827501

[9] Ali M, Nelson AR, Lopez AL, Sack DA. Updated global burden of cholera in endemic countries. PLoS Negl Trop Dis. 2015;9(6):e0003832. doi: 10.1371/journal.pntd.0003832 26043000 PMC4455997

[10] Shikanga O-T, Mutonga D, Abade M, Amwayi S, Ope M, Limo H, et al. High mortality in a cholera outbreak in western Kenya after post-election violence in 2008. Am J Trop Med Hyg. 2009;81(6):1085–90. doi: 10.4269/ajtmh.2009.09-0400 19996441

[11] Mremi IR, George J, Rumisha SF, Sindato C, Kimera SI, Mboera LEG. Twenty years of integrated disease surveillance and response in Sub-Saharan Africa: challenges and opportunities for effective management of infectious disease epidemics. One Health Outlook. 2021;3(1):22. doi: 10.1186/s42522-021-00052-9 34749835 PMC8575546

[12] Sauvageot D, Njanpop-Lafourcade B-M, Akilimali L, Anne J-C, Bidjada P, Bompangue D, et al. Cholera incidence and mortality in sub-Saharan African sites during multi-country surveillance. PLoS Negl Trop Dis. 2016;10(5):e0004679. doi: 10.1371/journal.pntd.0004679 27186885 PMC4871502

[13] Ganesan D, Gupta SS, Legros D. Cholera surveillance and estimation of burden of cholera. Vaccine. 2020;38 Suppl 1:A13–7. doi: 10.1016/j.vaccine.2019.07.036 31326254

[14] Olu OO, Usman A, Ameda IM, Ejiofor N, Mantchombe F, Chamla D, et al. The chronic cholera situation in Africa: why are African countries unable to tame the well-known lion?. Health Serv Insights. 2023;16:11786329231211964. doi: 10.1177/11786329231211964 38028119 PMC10647958

[15] ACAPS Analysis Hub. Ethiopia2014drivers of the cholera outbreak. 2024. 20240118_ACAPS_thematic_report_Ethiopia_drivers_of_cholera_outbreak.pdf

[16] World Health Organization African Regional Office. Technical guidelines for integrated disease surveillance and response in the African region. 2019. https://www.afro.who.int/publications/technical-guidelines-integrated-disease-surveillance-and-response-african-region-third-edition

[17] Wolfe CM, Hamblion EL, Dzotsi EK, Mboussou F, Eckerle I, Flahault A, et al. Systematic review of Integrated Disease Surveillance and Response (IDSR) implementation in the African region. PLoS One. 2021;16(2):e0245457. doi: 10.1371/journal.pone.0245457 33630890 PMC7906422

[18] Phalkey RK, Yamamoto S, Awate P, Marx M. Challenges with the implementation of an Integrated Disease Surveillance and Response (IDSR) system: systematic review of the lessons learned. Health Policy Plan. 2015;30(1):131–43. doi: 10.1093/heapol/czt097 24362642

[19] Ng’etich AKS, Voyi K, Kirinyet RC, Mutero CM. A systematic review on improving implementation of the revitalised integrated disease surveillance and response system in the African region: a health workers’ perspective. PLoS One. 2021;16(3):e0248998. doi: 10.1371/journal.pone.0248998 33740021 PMC7978283

[20] Fall IS, Rajatonirina S, Yahaya AA, Zabulon Y, Nsubuga P, Nanyunja M, et al. Integrated Disease Surveillance and Response (IDSR) strategy: current status, challenges and perspectives for the future in Africa. BMJ Glob Health. 2019;4(4):e001427. doi: 10.1136/bmjgh-2019-001427 31354972 PMC6615866

[21] Charnley GEC, Jean K, Kelman I, Gaythorpe KAM, Murray KA. Association between conflict and cholera in Nigeria and the Democratic Republic of the Congo. Emerg Infect Dis. 2022;28(12):2472–81. doi: 10.3201/eid2812.212398 36417932 PMC9707578

[22] World Bank. FY25 List of Fragile and Conflict-affected Situations. FCSListFY25.pdf.

[23] Cordes KM, Cookson ST, Boyd AT, Hardy C, Malik MR, Mala P, et al. Real-time surveillance in emergencies using the early warning alert and response network. Emerg Infect Dis. 2017;23(13):S131–S137. doi: 10.3201/eid2313.170446 PMC5711309 29155660

[24] World Health Organization. The status of health in the WHO African Region. State of health in the African Region.pdf

[25] Ohene S-A, Klenyuie W, Sarpeh M. Assessment of the response to cholera outbreaks in two districts in Ghana. Infect Dis Poverty. 2016;5(1):99. doi: 10.1186/s40249-016-0192-z 27802834 PMC5090876

[26] Omosigho PO, John OO, Musa MB, Aboelhassan YMEI, Olabode ON, Bouaddi O, et al. Stigma and infectious diseases in Africa: examining impact and strategies for reduction. Ann Med Surg (Lond). 2023;85(12):6078–82. doi: 10.1097/MS9.0000000000001470 38098545 PMC10718398

[27] Steinke A, Hövelmann S. Whose health matters: trust and mistrust in humanitarian crisis and global health interventions. Handbook of Global Health. Springer International Publishing. 2020;1–31. doi: 10.1007/978-3-030-05325-3_101-1

[28] Nations MK, Monte CM. “I’m not dog, no!”: cries of resistance against cholera control campaigns. Soc Sci Med. 1996;43(6):1007–24. doi: 10.1016/0277-9536(96)00083-4 8888470

[29] Cash RA, Narasimhan V. Impediments to global surveillance of infectious diseases: consequences of open reporting in a global economy. Bull World Health Organ. 2000;78(11):1358–67. 11143197 PMC2560626

[30] Kraemer MUG, Tsui JL-H, Chang SY, Lytras S, Khurana MP, Vanderslott S, et al. Artificial intelligence for modelling infectious disease epidemics. Nature. 2025;638(8051):623–35. doi: 10.1038/s41586-024-08564-w 39972226 PMC11987553

[31] Jiao Z, Ji H, Yan J, Qi X. Application of big data and artificial intelligence in epidemic surveillance and containment. Intell Med. 2023;3(1):36–43. doi: 10.1016/j.imed.2022.10.003 36373090 PMC9636598

[32] Bardosh KL, de Vries DH, Abramowitz S, Thorlie A, Cremers L, Kinsman J, et al. Integrating the social sciences in epidemic preparedness and response: a strategic framework to strengthen capacities and improve Global Health security. Global Health. 2020;16(1):120. doi: 10.1186/s12992-020-00652-6 33380341 PMC7772799

[33] Olu OO. The ebola virus disease outbreak in West Africa: a wake-up call to revitalize implementation of the international health regulations. Front Public Health. 2016;4:120. doi: 10.3389/fpubh.2016.00120 27376056 PMC4899437

[34] Belaid L, Sarmiento I, Dimiti A, Andersson N. Community participation in primary healthcare in the South Sudan Boma Health Initiative: a document analysis. Int J Health Policy Manag. 2022;11(12):2869–75. doi: 10.34172/ijhpm.2022.6639 35418007 PMC10105198

[35] Talisuna A, Yahaya AA, Rajatonirina SC, Stephen M, Oke A, Mpairwe A, et al. Joint external evaluation of the International Health Regulation (2005) capacities: current status and lessons learnt in the WHO African region. BMJ Glob Health. 2019;4(6):e001312. doi: 10.1136/bmjgh-2018-001312 31798983 PMC6861072

