# Supplementary material for: Unmasking and tackling the underestimation of the cholera burden in Africa: A viewpoint
Source: PLoS Negl Trop Dis. 2025 Jun 5;19(6):e0013128. doi: 10.1371/journal.pntd.0013128 (PMC12140239; doi:10.1371/journal.pntd.0013128)
Supplement: S1 File — (DOCX) [file pntd.0013128.s001.docx]

**Literature Search Strategy**

The evidence provided in this viewpoint article is based on the extensive practical field experiences of the authors in managing cholera outbreaks in Africa which was corroborated by the existing scientific literature on cholera underreporting. The literature used was obtained by conducting general searches in the main databases such as Pubmed, Google Scholar, African Journals Online, and African Index Medicus using the search term ("cholera" OR "underestimation" OR "under reporting") AND ("africa" OR "sub-saharan africa"). Forward and backward searches of all identified literature were then conducted. In the backward search for literature, the reference lists of the studies found using the above search term were reviewed while the forward search for references involved the review of the articles which had cited the relevant studies.
